# Astrocytes profiling in acute hepatic encephalopathy: Possible enrolling of glial fibrillary acidic protein, tumor necrosis factor-alpha, inwardly rectifying potassium channel (Kir 4.1) and aquaporin-4 in rat cerebral cortex

**DOI:** 10.3389/fncel.2022.896172

**Published:** 2022-08-17

**Authors:** Dalia Mahmoud Abdelmonem Elsherbini, Fatma M. Ghoneim, Eman Mohammed El-Mancy, Hasnaa Ali Ebrahim, Mohamed El-Sherbiny, Mohamed El-Shafey, Rasha Hamed Al-Serwi, Nehal M. Elsherbiny

**Affiliations:** ^1^Department of Clinical Laboratory Sciences, College of Applied Medical Sciences, Jouf University, Sakaka, Saudi Arabia; ^2^Department of Anatomy, Faculty of Medicine, Mansoura University, Mansoura, Egypt; ^3^Department of Histology and Cell Biology, Faculty of Medicine, Mansoura University, Mansoura, Egypt; ^4^Deanship of Common First Year, Jouf University, Sakaka, Saudi Arabia; ^5^Department of Zoology, Faculty of Women for Arts, Science and Education, Ain Shams University, Cairo, Egypt; ^6^Department of Basic Medical Sciences, College of Medicine, AlMaarefa University, Riyadh, Saudi Arabia; ^7^Department of Physiological Sciences, Fakeeh College for Medical Sciences, Jeddah, Saudi Arabia; ^8^Department of Basic Dental Sciences, College of Dentistry, Princess Nourah bint Abdulrahman University, Riyadh, Saudi Arabia; ^9^Department of Pharmaceutical Chemistry, Faculty of Pharmacy, University of Tabuk, Tabuk, Saudi Arabia; ^10^Department of Biochemistry, Faculty of Pharmacy, Mansoura University, Mansoura, Egypt

**Keywords:** GFAP, inwardly rectifying potassium channel, brain edema, aquaporin-4, TNFα

## Abstract

Hepatic encephalopathy (HE) is a neurological disarray manifested as a sequel to chronic and acute liver failure (ALF). A potentially fatal consequence of ALF is brain edema with concomitant astrocyte enlargement. This study aims to outline the role of astrocytes in acute HE and shed light on the most critical mechanisms driving this role. Rats were allocated into two groups. Group 1, the control group, received the vehicle. Group 2, the TAA group, received TAA (300 mg/kg) for 3 days. Serum AST, ALT, and ammonia were determined. Liver and cerebral cortical sections were processed for hematoxylin and eosin staining. Additionally, mRNA expression and immunohistochemical staining of cortical GFAP, TNFα, Kir4.1, and AQP4 were performed. Cortical sections from the TAA group demonstrated neuropil vacuolation and astrocytes enlargement with focal gliosis. GFAP, TNFα, and AQP4 revealed increased mRNA expression, positive immunoreactivity, and a positive correlation to brain water content. In contrast, Kir 4.1 showed decreased mRNA expression and immunoreactivity and a negative correlation to brain water content. In conclusion, our findings revealed altered levels of TNFα, Kir 4.1, GFAP, and AQP4 in HE-associated brain edema. A more significant dysregulation of Kir 4.1 and TNFα was observed compared to AQP4 and GFAP.

## Introduction

Hepatic encephalopathy (HE) and brain edema are serious consequences of acute liver failure (ALF). Cerebral derangement in subjects with ALF might proceed from cerebral impairment to a coma in a short time (Butterworth, 2009). Thioacetamide (TAA) is commonly used to experimentally induce HE following the International Society for HE and Nitrogen Metabolism (ISHEN) instructions (El-Ghazaly et al., 2020). The model simulates human acute progressive hepatic disarrays with concurrent brain implication (Butterworth, 2009). TAA induces hepatocellular necrosis, as well as lymphocyte infiltration in the absence of cholestasis. Additionally, the TAA model has been used to explain alterations in the central nervous system (CNS) functioning in HE (Guerit et al., 2009). Hyperammonemia is considered one of the most severe pathophysiologic mechanisms promoting cerebral edema in HE. Because a malfunctioning liver is unable to remove blood ammonia, its plasma and brain standards significantly increase (Sepehrinezhad et al., 2020).

Astrocytes constitute about 33% of the brain volume and are the prevailing glia in the brain (Sofroniew and Vinters, 2010). Astrocytes significantly contribute to the nervous system homeostasis *via* interlinking with neurons, capillary endothelial cells, and other glia cells (Sepehrinezhad et al., 2020). Various factors have been implicated in mediating astrocyte swelling. Astrocyte swelling and volume modulation are complicated procedures with many contributors (Lafrenaye and Simard, 2019). Inflammation has been documented to provoke astrocyte swelling. Indeed, ammonia promotes astrocyte swelling in astrocyte culture *in vitro* and is exacerbated by adding proinflammatory cytokines like tumor necrosis factor-alpha (TNFα), interleukins (IL)-1β, and IL-6 (Rama Rao et al., 2010b; Sepehrinezhad et al., 2020). Astrocytes respond to brain tissue derangement (whether it be due to injury, infection, or disease) by exhibiting astrogliosis, a process involving the overexpression of GFAP, proliferation, and cell body enlargement (Stevens and Halliday, 2014). The more severe the insults, the more reactive astrogliosis leading to glial scar formation (Schreiner et al., 2013).

ALF alters astrocytes’ ion homeostasis resulting in an extra-intracellular osmolar imbalance. The process of restoring osmotic equilibrium is associated with the entrance of water into cells *via* aquaporin water channels (AQPs). AQP4 is particularly abundant in the brain, notably in astrocytes, among numerous aquaporins (Rama Rao et al., 2010b). Indeed, AQP4-mediated osmotic balance restoration in different neurological illnesses, such as ischemic stroke, trauma, and tumors, leading to brain edema (Bloch and Manley, 2007; Zador et al., 2009). Additionally, in hyperammonemic brain, astrocyte membranes show high permeability to K^+^ ions, resulting in a hyperpolarized resting membrane potential and low input membrane resistance. Kir 4.1 inward rectifying K^+^ channel is the major player mediating these events (Murphy et al., 2017). Astrocytes may in turn downregulate Kir 4.1 channel gene expression to minimize ammonium absorption at the brain vessels and safeguard the brain due to their possible permeability for ammonium ions. Downregulation of these channels, though beneficial to the brain in terms of ammonia intake, would subsequently disrupt K^+^ and water homeostasis, possibly resulting in brain edema owing to water preservation and extensive neuronal depolarization due to greater extracellular K^+^ levels (Lichter-Konecki, 2008). Furthermore, previous studies reported that HE accompanied changes seen in astrocytes cannot easily be defined as swelling only but also astrogliosis (Hadjihambi, 2017).

Therefore, this study aims to outline the astrocytic role in acute HE, shedding light on the most important mechanisms driving this role. This is the first study to assess the role of GFAP, TNFα, Kir 4.1, and AQP4 in astrocytes in rat cerebral cortex in TAA models of ALF and HE.

## Materials and methods

### Sample size calculation

The G*Power program for Windows (version 3.1.9.7) was used to compute the sample size according to the procedures based on the previous research (Pawa and Ali, 2004; Wang et al., 2011; Demirel et al., 2012; El-Ghazaly et al., 2020). We assumed that the means for the two groups would be (121.855 and 498.075) for AST, (46.797 and 275.8) for ALT, and (65.345 and 254.755) for Ammonia. Considering the standard deviation (SD) within groups of (15.572 and 91.94) for AST, (6.705, and 88.03) for ALT, and (12.12 and 32.65) for ammonia, effect size (*f*) would be 5.706 for AST, 3.668 for ALT, and 7.691 for ammonia. According to these assumptions, samples sizes for AST, ALT, and ammonia were 6, 6, and 4, respectively, attaining 90% power to detect these effect sizes at an alpha level of 5%, considering the smallest effect size (3.668), *T*-test design with independent two groups has sample sizes of three per group. The total sample of eight achieves a power of 90% using the *F*-test with a target significance level of 0.05.

### Drugs and reagents

Thioacetamide (TAA) (C2H5NS) (Cat. #: 163678), an organosulfur compound, was first recognized as a hepatotoxic agent in rats by Fitzhugh and Nelson (1948), and polyclonal rabbit anti-aquaporin-4 (AQP4) (Cat. #: ABN910) were ordered from Sigma Chemical Co. (St. Louis, MO, United States). Polyclonal rabbit/anti-rat Ki67 antibody (ab15580), rabbit polyclonal to Kir4.1/KCNJ10 (ab240876), and rabbit polyclonal to glial fibrillary acidic protein (GFAP) (ab7260) were ordered from Abcam Co., Cambridge, United Kingdom. Polyclonal rabbit/anti-rat TNF-α antibodies were ordered from Lab Vision Co., Fremont, CA, United States (Cat. #:PA1-40281).

### Animals

In this study, adult Wistar male rats aged 6–8 months, weighing 250–350 g were used. Animals were kept in a standard laboratory condition at 25 ± 1°C with a relative humidity of 55% and a regular 12 h light/dark cycle. Free access to standard laboratory chow and water was available for rats.

### Experimental design

Rats were allocated into two groups of eight rats each. Group 1, the control group (no treatment), simply received a vehicle (0.9% saline in 0.1 ml intraperitoneally (i.p.) for 3 days. TAA was administered to Group 2 for 3 days.

#### Animal model of acute liver failure

ALF was prompted by a single daily (i.p.) injection of the TAA (300 mg/kg) for 3 days, as previously reported (Hilgier et al., 1983; Rama Rao et al., 2010c) at 17 h. The time of drug application was determined according to Lawrence and Beesley (1988) to achieve hepatotoxicity of thioacetamide. TAA administrated rats were given 12.5 ml/kg of isotonic solution containing 5% dextrose and 0.45% saline with 20 mEq/l potassium chloride s.c. every 12 h to avoid hypoglycemia, as reported earlier by Zimmermann et al. (1989). Saline (vehicle used for TAA) was administrated to the normal control group. This model had been applied for over 25 years and revealed morphological and clinical aberrations comparable to humans. Furthermore, according to the Evans blue extravasation technique, the TAA model of ALF does not lead to blood–brain barrier disruption (Rama Rao et al., 2010b). Rats were sacrificed 60 h following the second injection of TAA. Before scarification, blood samples were collected from the retroorbital plexus for serum isolation. The liver and brain were dissected and processed for histological and immunohistochemical studies. Half of the brain was used for the assessment of water content. For the molecular study, about 20–40 mg of tissue samples from the cerebral cortex were snap-frozen in liquid nitrogen and used immediately for total RNA extraction and real-time qPCR (RT-qPCR) examination of the expressions of GFAP, TNFα, Kir4.1, and AQP4.

### Hepatic encephalopathy assessment

**FIGURE 1:**
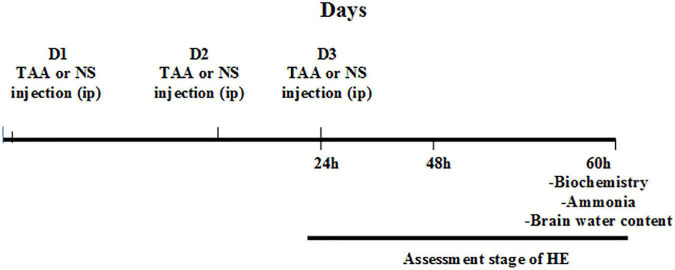
Experimental procedure. D1, the first day for thioacetamide (TAA) or normal saline (NS) injection; D2, the second day for TAA or NS injection; D3, the third day for TAA or NS injection 24 h: 24 h after the second TAA or NS injection, 48 h: 48 h after the second TAA or NS injection, and 60 h: 60 h after the second TAA or NS injection.

Clinical symptoms of rats and stages of HE in the different groups were assessed at different time intervals (24, 48, and 60 h) after injection of the second dose of TAA. Assessment of the HE stage was performed using a clinical and neuro-behavioral scale as previously described by Zimmermann et al. (1989). The diagram presenting the experimental procedure is shown in Figure 1.

### Assessment of serum aspartate aminotransferase, alanine aminotransferase, and ammonia levels

Aspartate aminotransferase (AST) and alanine aminotransferase (ALT) serum levels were determined using diagnostic kits purchased from the Biodiagnostic Co. (Cat. #: AT 1034 and AT 1045, respectively; Dokki, Giza, Egypt) by the application of method reported by Reitman and Frankel (1957). They were assessed by measuring the absorbance at 505 nm. Data obtained are represented as U/ml, where 1 unit is defined as 1 μmol of pyruvate formed under defined conditions per milliliter of serum.

For ammonia, blood was collected in a tube containing ethylenediaminetetraacetic acid (EDTA). The blood specimens were centrifuged immediately at 5–10°C to prevent hemolysis (Green, 1988). Serum ammonia level was detected by a direct enzymatic method previously described by Huizenga et al. (1994) using diagnostic kits purchased from the Biodiagnostic Co. (Cat. #: AM 1040; Dokki, Giza, Egypt). The stoichiometry of the reaction can be followed spectrophotometrically by NADH disappearance at 340 nm. Ammonia level was expressed as μmol/L.

### Real-time qPCR

Total RNA from rat cerebral cortex tissue samples (∼25 mg) was extracted using Trizol Reagent (Invitrogen) according to the manufacturer’s instructions and stored at –80°C. The concentration and purity of total isolated RNA were determined by NanoDrop spectrophotometry. Reverse transcription reaction for cDNA synthesis was performed with ∼200 ng total RNA using the Maxima First Strand cDNA Synthesis Kit (Thermo Scientific, United States; cat No. #K1641). The rat’s cerebral cortex mRNA expressions of GFAP, TNFα, Kir4.1, and AQP4 were quantified by the real-time PCR using the Applied Biosystem 7500, real-time PCR detection system (Life Technology, United States) with “HERAPLUS SYBR^®^ Green qPCR Master Mix” (2X) (Willowfort, United Kingdom; cat. No. WF10308001). Reaction mixtures were incubated for 10 min at 95°C, followed by 40 cycles of 15 s at 95°C and 30 s at 60°C. The primer sequences for rat GFAP (sense, 5′-CCTTGAGTCCTTGCGCGGCA-3′, antisense, 5′- TTGGCCCTCCTCCTCCAGCC-3′) (Liu C. et al., 2012), rat TNF-α: forward, 5′- AAT GGC CTC CCT CAT CAG TT-3′; reverse, 5′- CCA CTT GGT TTG CTA CGA -3′ (Abo El-khair et al., 2020), rat AQP4: forward, 5′-TGAATCCAGCTCGATCCTTTG-3′; reverse, 5′-TATCCAGTGGTTTTCCCAGTTTC-3′ (Shi et al., 2017), Kir4.1/Kcnj10 (forward: 5′-GTGACAGGCAAACTGCTTCA-3′; reverse: 5′-GGGCTATCAGAGGCTGTGTC-3′) (Liu Y. Z. et al., 2012). The primer sequences for rat β-actin (the control gene) were 5′- AAGATCCTGACCGAGCGTGG-3′ (Forward) and 5′- CAGCACTGTGTTGGCATAGAGG-3′ (Reverse) (He et al., 2018). The expression of the analyzed genes was normalized to that of the internal control gene, the β-actin, using the comparative ΔΔCT method.

### Liver and brain histopathological and immunohistochemical examination

Rats were anesthetized using 5% isoflurane inhalation and transcardially perfused with heparinized saline for 1 min, followed by fixation in 4% paraformaldehyde for 15 min (Park et al., 2018; Patkar et al., 2021). The dry weight method was used to assess cerebral edema. Animals were decapitated, and the brains were immediately removed. Half of the brain was weighed before and after 48 h of incubation in a 100°C oven. The water content of the brain samples was measured by the wet and dry weight method as follows (Zhan and Yang, 2006):


$$Water\;content(\%)=\frac{\left[Wet\;weight\;(mg)-Dry\;weight\;(mg)\right]}{Wet\;weight\;(mg)}\times 100\%$$

Half of the brains were kept in the same fixative for an additional 24 h. Coronal brain slices embedded in paraffin were stained with hematoxylin-eosin (Rama Rao et al., 2010c).

For the liver histological assessment, fixed specimens of the liver (left lobe) in 10% neutral buffered formalin were embedded in paraffin blocks and sliced in 4 μm thickness sections. The sections were stained with hematoxylin and eosin (H&E) and Masson’s trichrome stain (Bancroft and Gamble, 2008) and were photographed using a light microscope (BX-51; Olympus, Tokyo, Japan).

For immunohistochemistry, deparaffinated liver sections were stained with primary antibodies against Ki67 using the immunoperoxidase method (Saadoun et al., 2002). Cortical sections were stained with polyclonal rabbit/anti-rat antibodies using the immunoperoxidase method against GFAP, TNF-α, AQP4, and Kir4.1 (Masaki et al., 2010).

### Morphometric analysis

Hematoxylin and eosin-stained liver sections were examined and scored by two expert pathologists unaware of experimental procedures. The degree of hepatocyte necrosis was analyzed at 10 different high-power fields using a scale of 0–3 as previously described (Demirel et al., 2012): none, 0; mild, 1 (acidophilic bodies, ballooning degeneration, and/or scattered foci of hepatocellular necrosis in < 1/3 of lobules or nodules); moderate, 2 (involvement of 1/3–2/3 of lobules or nodules); severe, 3 (involvement of > 2/3 of lobules or nodules); and expressed as the mean within each slide. The extent of polymorphonuclear cell infiltration was also evaluated at 10 different high-power fields using a scale of 0–3 as follows: none, 0; mild, 1 (sprinkling of inflammatory cells in < 1/3 of portal tracts); moderate, 2 increased inflammatory cells in 1/3–2/3 of portal tracts); severe, 3 (dense packing of inflammatory cells in > 2/3 of portal tracts); and expressed as the mean within each slide. Morphometric measurements were carried out using the ImageJ^®^ software (Wayne Rasband NIH, Bethesda, MA, United States) (Schindelin et al., 2015), including at least two sections per animal (total = 16 sections/group), and from each section, three different non-overlapping fields were examined (48 fields/group). The following parameters were assessed: Number of the inflammatory cells/microscopic field in liver sections at a magnification of × 100. Mean diameter of central vein (μm)/16 sections/group, calibrations were performed with Image J using a straight-line tool at the appropriate calibration (pixel to μm ratio) at a magnification of × 100.

By using the following criteria, the scoring for the extent of liver fibrosis was assessed. Absence of any obvious fibrosis (0); the presence of fibrosis (1) showing the extension of collagen fibers from the central vein or portal triad to peripheral regions; mild fibrosis (2) indicating the presence of limited collagen fibers that extend without any formation of compartments; moderate fibrosis (3) showing presence of collagen fibers along with the development of “pseudo leaves”; and severe fibrosis (4) showing the presence of numerous collagen fibers along with stiffening of partial compartments as well as the development of “pseudo lobes” (Wang et al., 2019). Mean area % of collagen fiber content was measured in the Masson’s trichrome-stained sections at a magnification of × 100 using the ImageJ^®^ software, including at least two sections per animal (total = 16 section/group), and from each section, three different non-overlapping fields were examined (48 fields/group).

Hematoxylin and eosin-stained cortical sections were examined and scored by two experts. The percentage of cells with vacuoles, the number of astrocytes demarking gliosis and the area (%) of gliosis were determined at a magnification of × 100 using the ImageJ^®^ software, including at least two sections per animal (total = 16 section/group), and from each section, three different non-overlapping fields were examined (48 fields/group).

The mean surface area of astrocytes GFAP positive cells in cortical sections was measured at a magnification of × 400 using the ImageJ^®^ software, including at least two sections per animal (total = 16 section/group), and from each section, three different non-overlapping fields were examined (48 fields/group).

For the immunohistochemistry quantitative assessment, the Allred score was used (Fedchenko and Reifenrath, 2014; Iqbal and Buch, 2016), which is presented with a scale of 0–8 and quantified using the QuPath program (0.1.2) (Bancroft and Gamble, 2008). QuPath program depends on color grading for intensity in which blue color = negative, yellow color = mild intensity, orange color = intermediate intensity, and red color = strong intensity.

### Statistical analysis

Graphpad Prism, version 8, was used to analyze the data in this research. A one-way analysis of variance (ANOVA) and a Tukey’s *post-hoc* test were used. The independent samples’ *t*-test was applied to compare the means of two groups. Pearson’s test was used to perform a bivariate correlation. The *p*-value for significance was set at < 0.05. The data is provided as a mean ± SD.

## Results

### Biochemical and histopathological assessment of liver

Table 1 reveals significant increases in the serum levels of AST and ALT in the TAA group compared to the control group (*p* < 0.05). Scores of hepatocyte necrosis and polymorphonuclear leukocytes (PMNL) infiltration of the TAA group revealed a significant increase compared to the control group (*p* < 0.05).

**TABLE 1:** Biochemical parameters in experimental groups.

Variables	Groups	
	Control	TAA
AST (U/L)	144.72 ± 32.1	752.92 ± 75.2*
ALT (U/L)	51.94 ± 20.3	678.28 ± 43.5*
Ammonia (μmol/L)	50.81 ± 24.62	155.77 ± 49.22*

*Values are expressed as the mean ± SD.*

*p < 0.05 (between control and TAA group).

**FIGURE 2:**
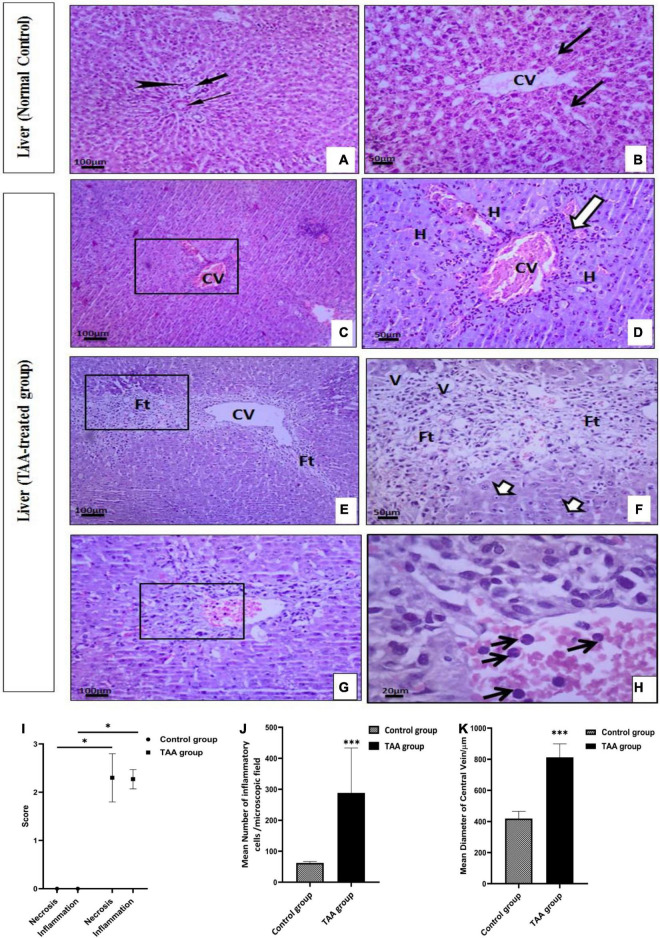
**(A–H)** Liver sections of control and TAA-treated groups stained with hematoxylin and eosin: **(A,B)** Control group. **(A)** Portal tract with normal portal venule (thin arrow), hepatic arteriole (arrowhead), and bile ductile (thick arrow). **(B)** Typical liver architecture with hepatic lobule-containing central vein (CV) and radiating hepatocytes cords with blood sinusoids (arrows) in between. **(C–H)** TAA-treated groups. **(C,D)** Dilation and congestion of CV (arrowheads) surrounded by inflammatory cell infiltrations (arrow) and hydropic degeneration of hepatocytes **(H)**. **(E,F)** Dilated CV is surrounded by massive fibrous tissue proliferation (Ft) associated with vacuolar degeneration (V) and nuclear pyknosis (short arrows). **(G,H)** Necrotic centrilobular area (rectangle) with inflammatory cell infiltration (arrow). X100 bar 100, X 400 bar 50 and X: 1,000 bar 20. **(I)** The graph represents hepatocyte necrosis and the extent of inflammatory cell infiltration. Data are expressed as mean ± SD. *p < 0.01 vs. control group. **(J)** The mean number of inflammatory cells/microscopic field. Data are displayed as mean ± SD. ^***^p < 0.001 vs. control group. **(K)** The mean diameter of the ventral vein (μm). Data are displayed as mean ± SD. ^***^p < 0.001 vs. control group.

**FIGURE 3:**
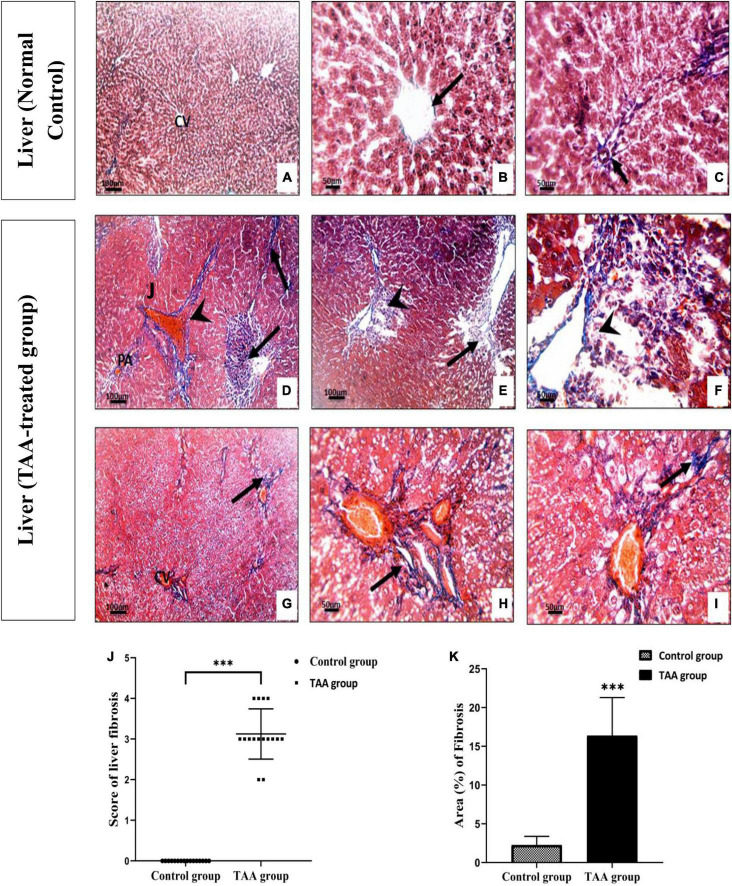
**(A–F)** Liver sections of control and TAA-treated groups stained with Masson’s trichrome. **(A–C)** Control group showing fine scarce collagen fibers (arrow) around central vein (CV) and portal areas (PA). **(D–I)** TAA-treated groups. **(D–F)** Excessive blue stained collagen deposition around the PA and dilated blood vessels (arrowhead) and severe fibrosis in liver tissue (arrow). **(G–I)** Extensive blue collagen deposition around congested CV extending to peripheral liver tissue (arrows) X100 bar 100, X 400 bar 50. **(J)** The scores of the degree of liver fibrosis. Data are expressed as mean ± SD. ^***^p < 0.001 vs. control group. **(K)** The area of collagen fibers in the liver (%). Data are displayed as mean ± SD. ^***^p < 0.001 vs. control group.

TAA administration caused dilation, congestion of blood vessels, increased inflammatory cell infiltrations, and hydropic degeneration of hepatocytes. It also induced dilation of the central vein and massive fibrous tissue proliferation (Figures 2, 3). Masson’s trichrome-stained sections were examined and scored for hepatic fibrosis. The sections showed strong deposition of coarse collagen fibers around the portal areas extending to hepatic lobular septa (score 3.13) in the TAA group compared to the control (Figure 3), which showed no fibrosis (score 0) except for fine scarce collagen around some portal areas and central vein. Furthermore, collagen deposition (mean area %) was significantly (*p* < 0.001) higher in the TAA group as showed by the quantitative analysis using the Image J software (Figures 3J,K).

**FIGURE 4:**
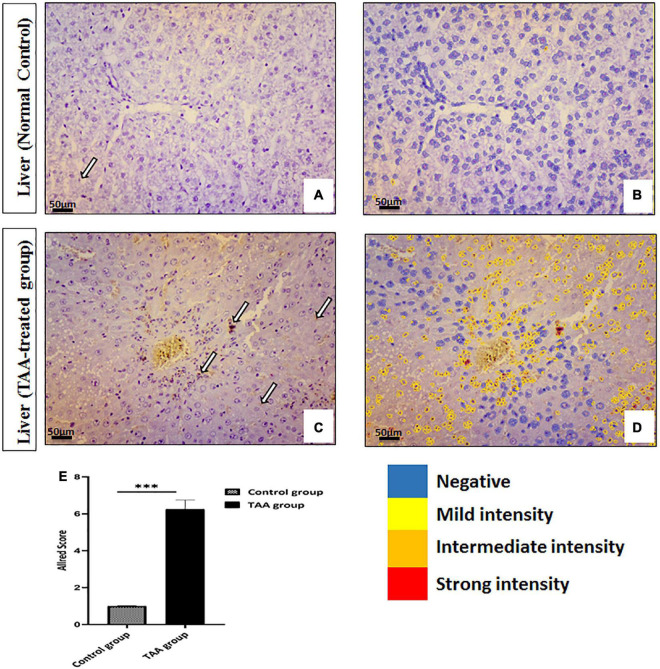
**(A–D)** Liver sections of control and TAA-treated groups stained with ki-67. **(A,B)** Control groups. **(A)** Very rare immunopositive hepatocytes (arrow). (B) Quantitative analysis of cells showing negativity and mild intensity. **(C,D)** TAA-treated groups. **(C)** An increased number of immunopositive nuclei of hepatocytes (arrows). **(D)** Quantitative analysis of cells showing different intensities varying from mild to strong. X: 400 bar 50. **(E)** Allred immunohistochemical scoring system. Data are expressed as mean ± SD. 0–1 (negative), 2–3 (mild), 4–6 (moderate), and 7–8 (strong). ^***^p < 0.001 vs. control group.

Microscopic examination of the immunostained liver specimens against Ki67 exhibited negative staining in the control group (Figures 4A,B). A moderate positive nuclear expression in the TAA group was noticed (Figures 4C,D). This expression was assessed by the Allred score that showed a positive reaction in the TAA group. Quantitate analysis showed increased nuclear expression when compared with the control group (Figures 4B,D,E).

### Hepatic encephalopathy staging

Table 2 shows HE stages for the study groups at different time intervals (24, 48, and 60 h) following injection of the second dose of TAA. The control group showed no evidence of encephalopathy. TAA-administrated rats developed encephalopathy progressively over the time course.

**TABLE 2:** Stages of hepatic encephalopathy (HE).

	Stages of HE					

	Number	0	I	II	III	IV
**24 h**						
Control group	8	8	0	0	0	0
TAA group	8	0	6	2	0	0
**48 h**						
Control group	8	8	0	0	0	0
TAA group	8	0	2	5	1	0
**60 h**						
Control group	8	8	0	0	0	0
TAA group	8	0	1	3	4	0

### Brain water content

**FIGURE 5:**
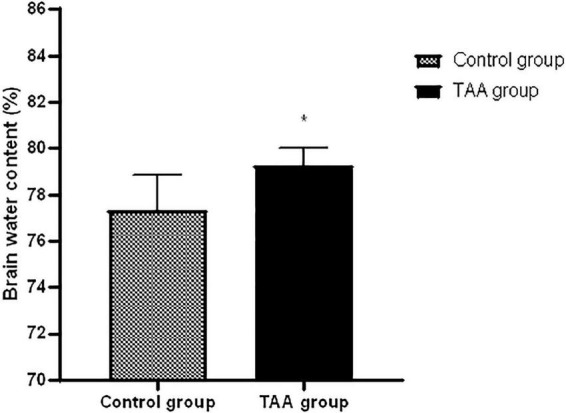
Mean ± SD values of brain water in treated groups. *p < 0.05 is significantly different in the TAA-treated group compared to the control group.

TAA-administrated rats showed a significantly higher brain water content than the corresponding control rats as shown in Figure 5 (*p* < 0.05).

### Gene expression of glial fibrillary acidic protein, tumor necrosis factor-alpha, Kir4.1, and anti-aquaporin-4

As shown in Table 3, the results of gene expression analysis showed a significant increase in the mRNA gene expression of brain GFAP, TNFα, and AQP4 with a concomitant decrease in the mRNA gene expression of brain Kir4.1 in the TAA-treated group compared to the control group (*p* < 0.05).

**TABLE 3:** mRNA expression of GFAP, TNF-α, AQP4, and Kir4.1 in cerebral cortex of control and TAA-treated groups.

	Group I (The control group)	Group II (TAA-treated group)
GFAP gene expression (2^–ΔΔCT^)	1 ± 0.02	1.91 ± 0.16*
TNF-α gene expression (2^–ΔΔCT^)	1 ± 0.02	2.12 ± 0.13*
AQP4 gene expression (2^–ΔΔCT^)	1 ± 0.03	2.41 ± 0.11*
Kir4.1 gene expression (2^–ΔΔCT^)	1 ± 0.03	0.61 ± 0.04*

*Significant compared to control group.

### Histopathological assessment of cerebral cortex

#### Hematoxylin and eosin-stained sections

**FIGURE 6:**
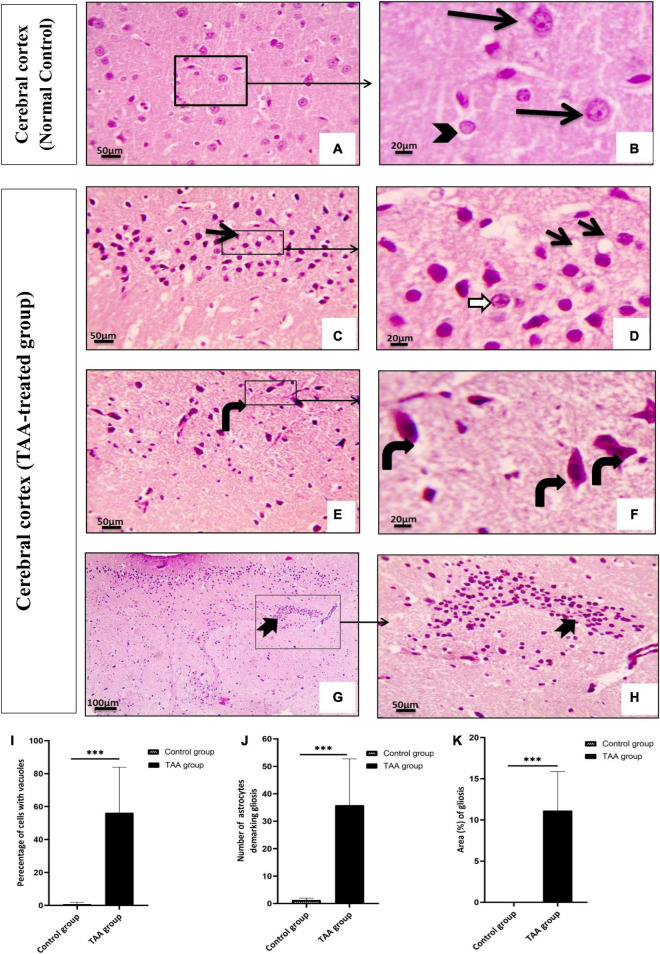
**(A–H)** The cerebral cortex of control and treated groups stained with **(H&E)**. **(A)** Control group exhibiting the normal texture of the neuropil between the neurons. **(B)** Higher magnifications of the control group showing normal pyramidal neurons with their large nuclei and prominent nucleoli, basophilic cytoplasm (black arrows), normal astrocytes having larger round nuclei with pale vesicular chromatin patterns and indistinct nucleoli (arrowhead). **(C–H)** Cerebral cortical sections from the TAA-treated group. **(C,D)** Neuropil vacuolation (short arrows) and astrocytes with enlarged and relatively clear nuclei (white arrows). **(E,F)** Severely affected neurons with prominent eosinophilic cytoplasm and nuclear pyknosis (curved arrows). **(G,H)** Focal gliosis (bifid arrow). X: 400 bar 50 and X: 1000 bar 20. **(I)** Quantitative analysis showing the percentage of cells with vacuoles. **(J)** Graph showing number of astrocytes demarking gliosis and **(K)** showing area percentage of gliosis. Values represent mean ± SD (^***^p < 0.001 vs. control group).

Examination of the cerebral cortex of the control group revealed the typical texture of the neuropil between the neurons. The pyramidal neurons were normal with large nuclei, prominent nucleoli, and basophilic cytoplasm. Astrocytes typically had larger round nuclei with pale vesicular chromatin patterns and indistinct nucleoli (Figures 6A,B). Cerebral cortical sections from the TAA-treated group showed neuropil vacuolation and astrocytes swelling with enlarged and relatively clear nuclei (Figures 6C,D,I). Neurons were severely affected with prominent eosinophilic cytoplasm and nuclear pyknosis (Figures 6E,F). Focal gliosis was evident in Figures 6G,H,J,K).

#### Immunohistochemistry-stained sections

**FIGURE 7:**
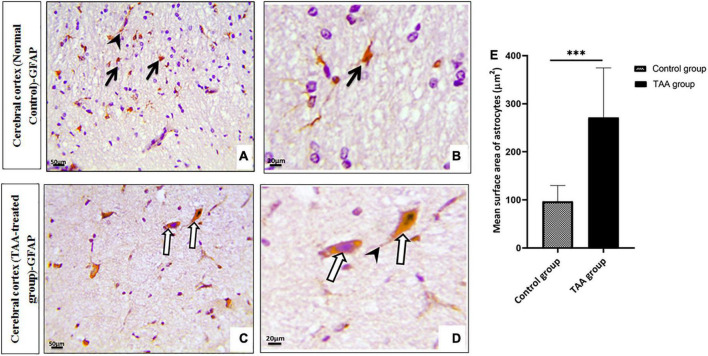
**(A–D)** Microscopic pictures of immunostained cerebral cortical sections against GFAP of control and treated groups. **(A,B)** Control group showing positively brown stained astrocytes (black arrows) with evident expression in their processes (arrowhead). **(C,D)** Cerebral cortical sections from the TAA-treated group showing hypertrophied astrocytes (white arrows) and processes (arrowhead). X: 400 bar 50 and X: 1000 bar 20. **(E)** Graph showing mean surface area of astrocytes. Values represent mean ± SD (^***^p < 0.001 vs. control group).

Microscopic pictures of immunostained cerebral cortical sections against GFAP revealed positively brown stained astrocytes of the control group (Figures 7A,B). Cerebral cortical sections from the TAA-treated group showed hypertrophied astrocytes (Figures 7C,D). The mean surface area of astrocytes was significantly increased in the TAA-treated group as compared with the control group (Figure 7E).

**FIGURE 8:**
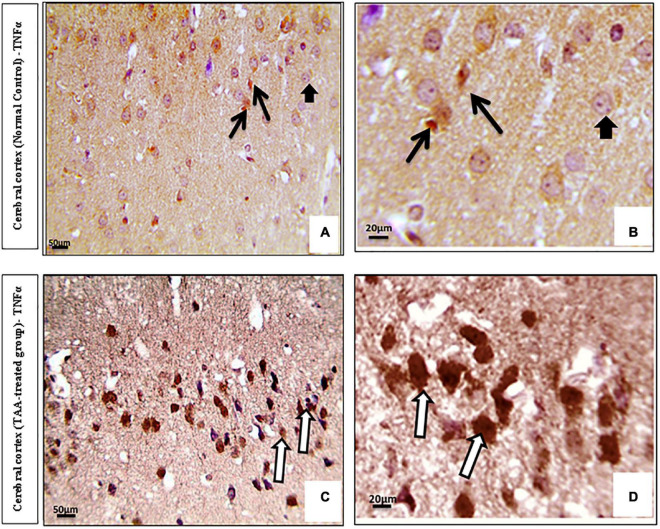
**(A–D)** Microscopic pictures of immunostained cerebral cortical sections against TNFα of control and treated groups. **(A,B)** Control group showing mild expression (black arrows) or negative staining (short arrows) in astrocytes. **(C,D)** Cerebral cortical sections from the TAA administrated group demonstrating strong positive brown astrocytic staining (area of gliosis) (white arrows). X: 400 bar 50 and X: 1000 bar 20.

Immunostained cerebral cortical sections against TNFα revealed mild expression or negative staining in astrocytes of the control group (Figures 8A,B). Cerebral cortical sections from the TAA-treated group showed intense positive brown astrocytic staining (area of gliosis) (Figures 8C,D).

**FIGURE 9:**
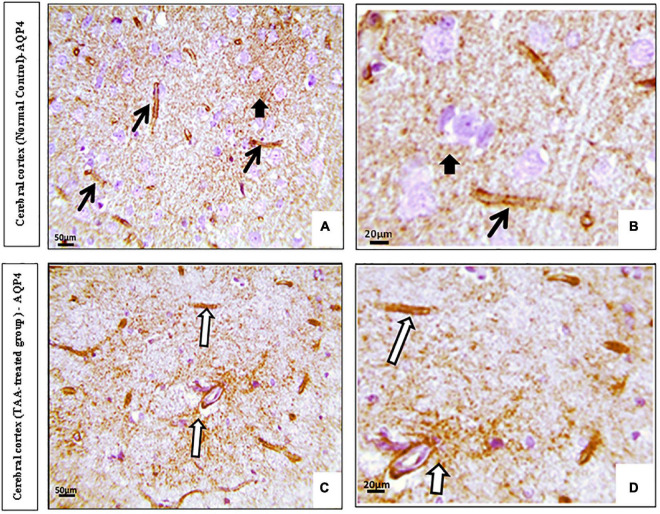
**(A,D)** Microscopic pictures of immunostained cerebral cortical sections against (AQP4) of control and treated groups. **(A,B)** Control group showing expression (black arrows) in the blood vessels and membranes of astrocytes (short arrow). **(C,D)** Cerebral cortical sections from the TAA-treated group showing increased positively brown stained blood vessels (white arrows). X: 400 bar 50 and X: 1000 bar 20.

Immunostained cerebral cortical sections against AQP4 revealed expression in the blood vessels and membranes of astrocytes of the control group (Figures 9A,B). Cerebral cortical sections from the TAA-treated group showed increased positively brown stained blood vessels (Figures 9C,D).

**FIGURE 10:**
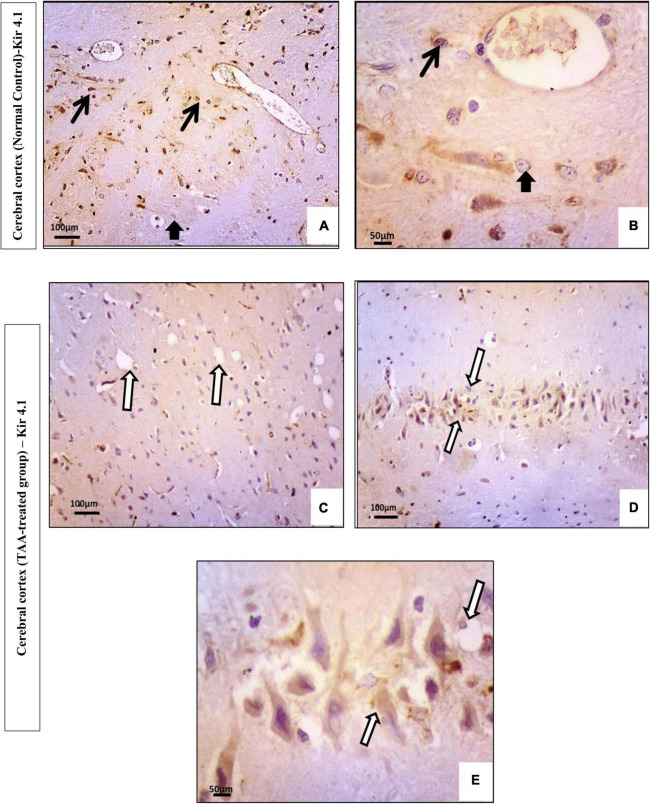
**(A–E)** Microscopic pictures of immunostained cerebral cortical sections against Kir4.1 of control and treated groups. **(A,B)** Control group showing expression in astrocytes near the blood vessels (black arrows) and negative expression in neurons (short arrow). **(C–E)** Cerebral cortical sections from the TAA-treated group. **(C)** Micrograph showing vacuolation and pyknotic nuclei of astrocytes with decreased expression (white arrows). **(D,E)** Hypertrophied astrocytes with moderated expression (arrowhead). X: 400 bar 50 and X: 200 bar 100.

Immunostained cerebral cortical sections against Kir4.1 revealed expression in astrocytes near the blood vessels and negative expression in neurons of the control group (Figures 10A,B). Cerebral cortical sections from the TAA-treated group showed vacuolation and pyknotic nuclei of astrocytes with decreased expression (Figure 10C). It revealed hypertrophied astrocytes with moderated expression (Figures 10D,E).

### Quantitative analysis

**FIGURE 11:**
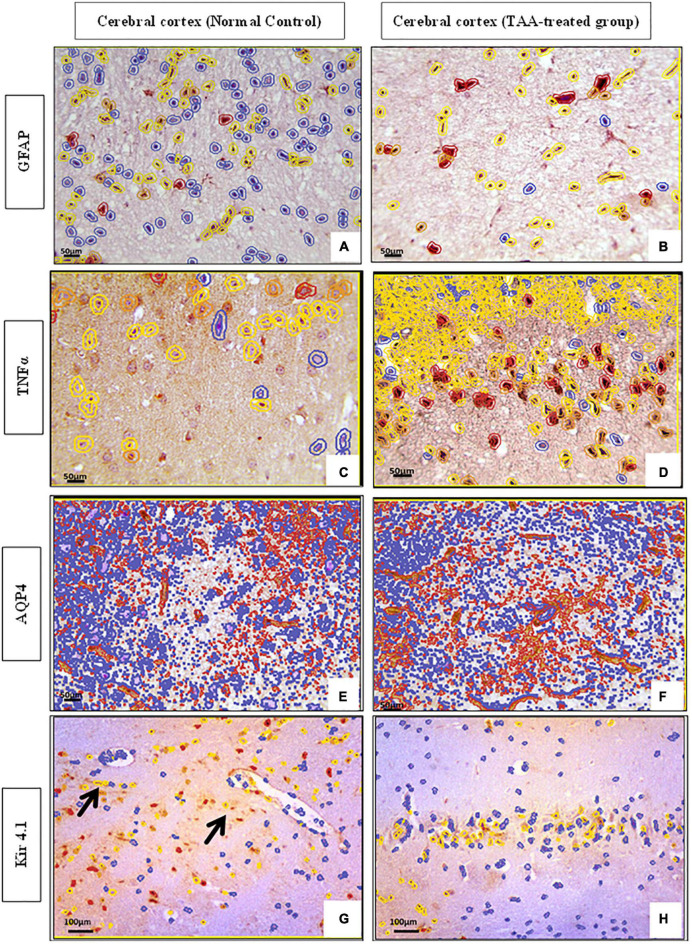
**(A–H)** QuePath Quantification of microscopic pictures of immunostained cerebral cortical sections of control and treated groups. **(A,B)** Cerebral cortical sections against (GFAP) showing increased intensity in TAA-treated group as indicated by an increase in the red color of astrocytes **(B)** when compared with control group **(A)**, which showed an increase in blue color. **(C,D)** Cerebral cortical sections against (TNFα) showing increased intensity in TAA-treated group as indicated by an increase in the yellow and red color of astrocytes **(B)** as compared with the Control group **(A)**, which showed a decrease in red and yellow color. **(E,F)** Cerebral cortical sections against (AQP4) showing increased intensity in TAA-treated group as indicated by an increase in orange and red color in the blood vessels and membranes of astrocytes **(B)** when compared with control group **(A)**, which displayed a decrease in red color. **(G,H)** Cerebral cortical sections against (Kir4.1) showing decreased intensity in TAA-treated group as indicated by a decrease in the orange and red color of astrocytes **(B)** as compared with the Control group **(A)**, which showed an increase in red color and yellow color in astrocytes, especially near blood vessels (black arrows). X: 400 bar 50 and X: 200 bar 100.

QuePath quantification of microscopic pictures of immunostained cerebral cortical sections was performed. Cerebral cortical sections against GFAP demonstrated increased intensity in the TAA-treated group as indicated by an increase in the red color of astrocytes (Figure 11B) as compared with the control group (Figure 11A), which showed an increase in blue color. Cerebral cortical sections against TNFα showed increased intensity in the TAA-treated group as indicated by an increase in the yellow and red color of astrocytes (Figure 11D) when compared with the control group (Figure 11C), which displayed a decrease in red and yellow colors. Cerebral cortical sections against AQP4 revealed increased intensity in TAA-treated group as indicated by an increase in orange and red color in the blood vessels and membranes of astrocytes (Figure 11F) as compared with the control group (Figure 11E), which showed a decrease in red color. Cerebral cortical sections against Kir4.1 displayed a decreased intensity in TAA-treated group as indicated by a reduction in the orange and red colors of astrocytes (Figure 11H) when compared with the control group (Figure 11G), which exhibited an increase in red and yellow colors in astrocytes, especially near blood vessels.

**FIGURE 12:**
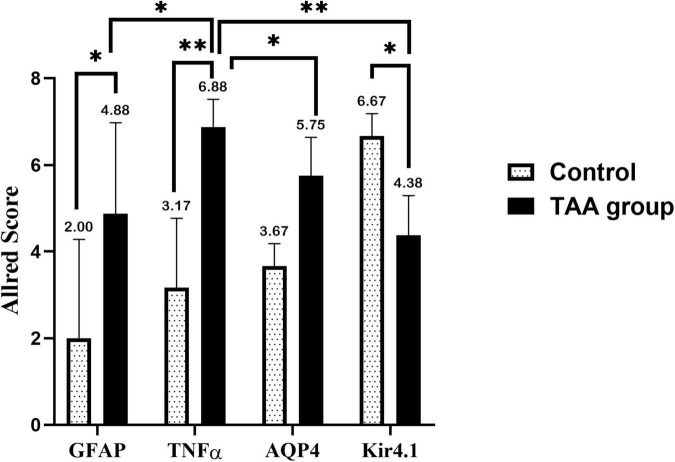
Allred immunohistochemical scoring system. Data are expressed as mean ± SD. 0–1 (negative), 2–3 (mild), 4–6 (moderate), and 7–8 (strong). Strong expression of TNFα and AQP4 is noticed in the TAA-treated group. Allred score of TNFα is higher as compared with AQP4. On the other hand, the Allred score of Kir 4.1 in the control group is significantly high compared with the TAA-treated group. *p < 0.05, ^**^p < 0.01.

This expression was evaluated by the Allred score (Figure 12). Data are expressed as mean ± SD. Strong expression of TNF-α and AQP4 was noticed in the TAA-treated group. The Allred score of TNFα was higher as compared with AQP4. On the other hand, the Allred score of Kir 4.1 in the control group was significantly higher as compared to the TAA-treated group.

**FIGURE 13:**
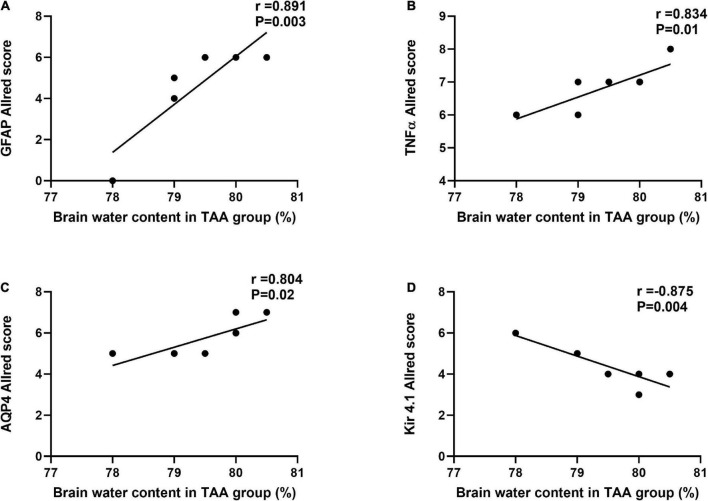
Correlations between Allred score of GFAP **(A)**, TNF-α **(B)**, AQP4 **(C)**, and Kir 4.1 **(D)** and brain water content in TAA administrated group. Significant positive correlations were noted for GFAP, TNFα, and AQP4, while Kir 4.1 was negatively correlated.

Figure 13 shows a correlation between the Allred score of GFAP, TNFα, AQP4, Kir 4.1, and brain water content in the TAA-treated group. Interestingly, Allred scores of GFAP, TNFα, and AQP4 (Figures 13A–C), respectively, were significantly positively correlated with brain water content in TAA-administrated rats. In contrast, the Allred score of Kir 4.1 (Figure 13D) was negatively correlated with brain water content in TAA-administrated rats.

## Discussion

**FIGURE 14:**
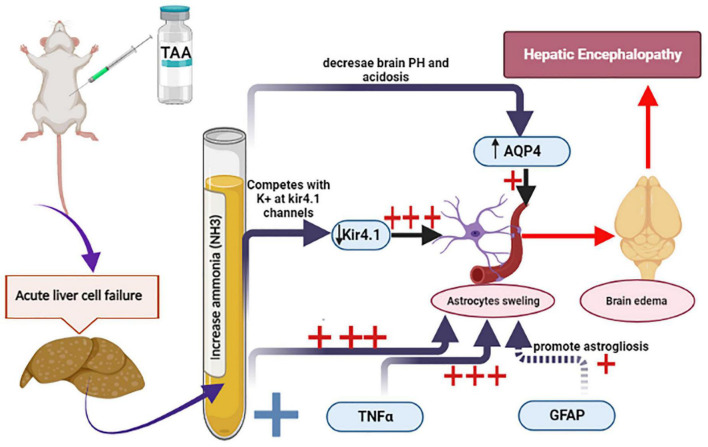
Schematic diagram summarizes the current experiment and the most important mechanisms involved in astrocytes swelling. Increased ammonia level and TNFα and decreased Kir 4.1 expression were the most prominent factors (+++) as compared with GFAP and AQP4 (+).

The main purpose of this research work was to clarify the role of astrocytes in acute HE and to outline the most important mechanisms involved. For this purpose, GFAP, TNFα and AQP4, and Kir 4.1 in astrocytes were assessed. This work showed that cerebral cortical sections from the TAA-treated group demonstrated neuropil vacuolation and astrocytes with enlarged and relatively clear nuclei. Immunostained cerebral cortical sections against GFAP and Kir 4.1 revealed hypertrophied astrocytes. Dysregulation of TNFα and Kir 4.1 was more significant. Indeed, a marked increase in mRNA levels of TNFα and a decrease in mRNA level of Kir4 in astrocytes were observed as compared with GFAP and AQP4. The results of the current study are summarized in Figure 14.

The experimental rat model of acute hepatic failure and HE produced by TAA is a reliable and acceptable HE model (Obara-Michlewska et al., 2011; Grant et al., 2018). TAA in this study was administrated for 3 days as a single daily (i.p.) injection at 17:00 h. The time of application was chosen to achieve TAA hepatotoxicity as it was reported by Lawrence and Beesley (1988) to be related to diurnal variations in flavin monooxygenase activity. They also stated that this enzyme can carry out the critical conversion of thioacetamide-S-oxide to thioacetamide dioxide from which polar intermediates that bind irreversibly to cellular macromolecules are derived. Flavin monooxygenase activity is believed to peak at 17.00 h. In this work, we developed TAA-induced ALF in rats and showed that all the rats presented typical HE symptoms, including lethargy, mild ataxia, and lack of spontaneous movement. In addition, rats administrated with TAA induced severe liver damage in the form of liver dysfunction as evident by biochemical tests, dilation, congestion of blood vessels, increased inflammatory cell infiltrations, and hydropic degeneration of hepatocytes as shown by histological assessment. Moreover, immunostained liver sections against Ki67 displayed moderate positive nuclear expression in the TAA group. Similar results were previously recorded (Miranda et al., 2010; Grant et al., 2018; Alican et al., 2019; El-Ghazaly et al., 2020).

In this study, TAA-administrated rats showed significantly higher brain water content as compared to control rats. Cerebral edema is a potentially fatal sequel of ALF. This issue could result in brain herniation and increased intracranial pressure. Hyperammonemic states and neuroinflammation disrupt glial functioning and result in neuronal damage (Sepehrinezhad et al., 2020). The exact cause of cerebral edema in ALF is yet to be understood. In mice, an acute administration of TAA raises brain water content and causes cerebral edema (Grant et al., 2018). Neuroinflammation and ammonia-dependent events might be involved in increased water content in TAA-treated rats (Heidari et al., 2016; Jia et al., 2020). This was ascertained in our study by increased plasma ammonia level along with increased TNFα expression in astrocytes. Moreover, the Allred score of TNFα was significantly positively correlated with brain water content in TAA-administrated rats. A similar study conducted by Wang et al. (2011) concluded that progressive brain edema was associated with significant increases in brain proinflammatory cytokines. Excessive formation of proinflammatory factors in the early stage of HE may exaggerate brain edema, participating in the development of HE. These data support the hypothesis that proinflammatory cytokine accumulated in the brain is linked with the evolution of brain edema in HE.

Astrocytes play a significant role in CNS homeostasis *via* pH regulation, K^+^ spatial buffering, lactate supply to neurons and ATP production, neuronal glutathione production, osmolytes regulation, and glutamate production (Allaman et al., 2011). Their surface membranes have different receptors, transporters, channels, and gap junctions, representing a high level of importance (Sepehrinezhad et al., 2020).

Astrocytes’ swelling is the major contributor to brain edema that may induce brain herniation and death by promoting hypertension. Different factors may drive astrocytes’ swelling. Nevertheless, the precise molecular pathways of astrocytes’ swelling are not entirely known (Sepehrinezhad et al., 2020). Mechanisms of this swelling in our study could be first attributed to increased ammonia plasma level and inflammatory signaling that could worsen the condition in hyperammonemia by reinforcing effects on the astrocytes swelling (Sepehrinezhad et al., 2020). In line, astrocyte culture containing ammonia showed astrocyte swelling exacerbated by proinflammatory cytokines TNFα and interleukins (Rama Rao et al., 2010a). Indeed, neuroinflammation in the hyperammonemic brain in this study was evident by increased TNFα and GFAP gene expression and immunostaining in astrocytes. Inflammatory cytokines, such as TNFα, aggravate ammonia-induced astrocyte swelling in an oxidative stress-dependent manner (Lachmann et al., 2013). Previous studies reported that GFAP^+^ cells as a marker of neuroinflammation had increased two times in the TAA group as compared to the control group. Administration of TAA augmented GFAP expression as an index for astrogliosis in the mice cerebellum and hippocampus (Sepehrinezhad et al., 2021). However, earlier studies showed controversial results; postmortem immunohistochemistry study of brain sections of patients with HE showed GFAP decline in the cerebral cortex, thalamus, basal ganglia, and subcortical white matter (Sobel et al., 1981). Furthermore, a decline in GFAP had been observed *in vitro* in astrocytes treated with ammonia (Norenberg et al., 1990). This discrepancy may prove that GFAP has a great role in both astrocyte swelling and astrogliosis. This was evident in our work by increased astrogliosis. The increase in GFAP expression in our study and its role in astrocytic swelling could be explained by the difference in the time course of observations on astrocytic plasticity; it could reflect the volume transfer during regulatory volume decrease (RVD). A previous study conducted by Wang et al. (2013a) reported an initial increase in GFAP expression followed by a decline in its expression in the hypothalamus. They added that alteration in GFAP expression was accompanied by simultaneous alteration in glutamine synthetase expression and redistribution of this enzyme toward peripheral processes (Wang et al., 2013a,b). It has been well established that astrocytic morphological plasticity is closely associated with the cytoplasmic fluid transfer between astrocyte soma and processes around neurons and that an increase in somatic GFAP staining or volemic expansion often results from RVD occurring at the processes (Wang and Parpura, 2018; Li et al., 2020). Through this GFAP plasticity, astrocytes can adaptively exert their influence on the activity of neurons *via* differently localizing their functional proteins and changing the neurochemical environment (Wang and Parpura, 2018). The high GFAP morphological plasticity could be attributed to its quick changes in assembling and polymerizing states in response to environmental challenges (Li et al., 2020). These previously mentioned observations explain excitotoxicity developed in the TAA model of HE and presented in large astrocytic somata and increased GFAP staining with volume transfer between astrocytic compartments during stages of encephalopathy.

Another factor involved in astrocyte swelling is AQP4. Our study showed increased gene and protein expression of AQP4 in the blood vessels and membranes of astrocytes in the TAA-treated group. Also, a positive correlation between the Allred score of AQP4 expression and brain water content was demonstrated. AQP4 channels in astrocytes and other brain cells are primarily responsible for transmitting water molecules across the membrane (Agre et al., 2004). In hypoosmotic states, astrocytes prevent excessive swelling by excreting osmolytes, the process is known as regulatory volume decline (Ordaz et al., 2004). As a result of the malfunction of this system, astrocytes are more liable to swelling than brain cells due to their exceptionally high expression of AQP4 (Papadopoulos and Verkman, 2013). The AQP4 channel expression in HE animal models is controversial. For instance, enhanced expression and immunostaining of AQP4 were observed on the end feet membranes of perivascular astrocytes in the cerebral cortex of ALF patients and in TAA-induced ALF in rats, which contribute to cerebral edema (Rama Rao et al., 2010b; Thumburu et al., 2014). The decline of intracellular pH enhances cellular swelling and the expression of AQP4 by astrocytes. This is achieved by ammonia that may enhance acidosis by accumulating lactate in astrocytes (Morishima et al., 2008). Another investigation on ALF rats found that AQP4 expression did not coincide with the degree of cerebral edema or hyperammonemia (Wright et al., 2010). This discrepancy could be explained by different conditions accompanied by cerebral edema. Cytotoxic edema is characterized by cell swelling in astrocytes while the BBB stays intact. However, in vasogenic edema, the permeability of the BBB increases, and there is a net outflow of water and blood components into the extracellular space (Sepehrinezhad et al., 2021).

The results of this study showed vacuolation and pyknotic nuclei of astrocytes with decreased expression of Kir 4.1 in the TAA-treated group in some sections, while others revealed hypertrophied astrocytes with moderate expression. Also, the Allred score of Kir 4.1 in the control group is significantly high as compared to the TAA-treated group. In addition, the Allred score of Kir 4.1 showed a significant negative correlation with brain water content in TAA-administrated rats. Additionally, Kir 4.1 mRNA gene expression was markedly decreased in the TAA-treated group compared to the control. The Kir 4.1 inward rectifying K^+^ channel plays a key role in modulating the high permeability of astrocyte membranes to K^+^ ions. The passive transport of K^+^ *via* Kir 4.1 channels has been proposed as being crucial for K^+^ spatial buffering (Murphy et al., 2017). The AQP4 water channel and Kir4 have been linked both physically and functionally in several studies (Strohschein et al., 2011). The co-localization of Kir 4.1, Kir 5.1, Cx43, and AQP4 in the endfeet of astrocytes at capillaries and the pia suggests that modulation of extracellular K^+^ levels may be linked to water transport (Hibino et al., 2004; Haj-Yasein et al., 2011). Furthermore, several investigations have shown a decrease in Kir 4.1 expression coupled with astrocyte swelling after edema-inducing diseases (Milton and Smith, 2018). On the other hand, others reported no changes in astrocyte swelling in the lack of Kir 4.1 (Larsen et al., 2014). The ammonia ion has been proven to imitate and replace the K^+^ ion at channels, receptors, and transporters (Holm et al., 2005). The alteration in the channels expression that assists K^+^ and water homeostasis in the brain might be a direct outcome of enhanced ammonium ions in the cerebrum. Downregulation of these channels, which would benefit the brain in terms of ammonia absorption, would interfere with K^+^ and water homeostasis, potentially leading to cerebral edema as a result of water retention and extensive neuronal depolarization induced by increased extracellular K^+^ levels (Lichter-Konecki, 2008).

## Conclusion

In conclusion, this study showed the implication of astrocytes swelling in HE. Results also showed that TNFα and Kir 4.1 were more significantly altered in astrocytes compared with GFAP and AQP4. TNFα expression in the cerebral cortex was increased in TAA administrated group which might be attributed to the hyperammonia state. On the other hand, Kir 4.1 expression in astrocytes was significantly decreased in TAA administrated group. The brain damage produced by acute hyperammonemic encephalopathy may be triggered by an astrocyte’s defensive regulatory response to high blood ammonia levels, downregulating channels that ammonia may utilize to reach the brain. This downregulation might have a deleterious impact on water retention and extracellular potassium increase, resulting in cerebral edema.

## Data availability statement

The original contributions presented in this study are included in the article/Supplementary material, further inquiries can be directed to the corresponding authors.

## Ethics statement

The research design, applied in our study, meets Animal Research: Reporting of *in vivo* Experiments (ARRIVE) guidelines. All the experimental procedures were carried out according to the Guidelines for the Care and Use of Laboratory Animals established by the National Institutes of Health of the United States (NIH) publication (No. 85-23, 1996) and according to the Animal Care and Use Committee at the Faculty of Medicine, Mansoura, Egypt. The protocol of the study was approved by the Mansoura Medical Research Ethics Committee (Approval No. 21.06.1353).

## Author contributions

All authors listed have made a substantial, direct, and intellectual contribution to the work, and approved it for publication.
